# Clinical significance and therapeutic value of glutathione peroxidase 3 (GPx3) in hepatocellular carcinoma

**DOI:** 10.18632/oncotarget.2549

**Published:** 2014-10-11

**Authors:** Xiang Qi, Kevin Tak Pan Ng, Qi Zhou Lian, Xiao Bing Liu, Chang Xian Li, Wei Geng, Chang Chun Ling, Yuen Yuen Ma, Wai Ho Yeung, Wen Wei Tu, Sheung Tat Fan, Chung Mau Lo, Kwan Man

**Affiliations:** ^1^ Department of Surgery, Centre for Cancer Research, The University of Hong Kong, Pokfulam, Hong Kong, China; ^2^ Department of Medicine, The University of Hong Kong, Pokfulam, Hong Kong, China; ^3^ Department of Paediatrics & Adolescent Medicine, The University of Hong Kong, Pokfulam, Hong Kong, China

**Keywords:** GPx3, HCC, hiPSC-MSCs, Tumor suppressor gene, Prognosis

## Abstract

Aims: We aimed to investigate the clinical significance of GPx3 in hepatocellular carcinoma (HCC) and to characterize its tumor suppressive role.

Methods: HCC patients (113) who underwent hepatectomy were recruited to examine the clinical relevance of GPx3. The tumor suppressive role of GPx3 was studied by administration of recombinant GPx3 (rGPx3) or over-expression of GPx3 in HCC cells *in vitro* and *in vivo*. The therapeutic value of GPx3 for HCC was further investigated using human induced pluripotent stem cell derived mesenchymal stem cells (hiPSC-MSCs) as its delivery vehicle.

Results: Down-regulation of GPx3 significantly correlated with advanced tumor stage (*P* = 0.024), venous infiltration (*P* = 0.043) and poor overall survival (*P* = 0.007) after hepatectomy. Lower plasma GPx3 in HCC patients was significantly associated with larger tumor size (*P* = 0.011), more tumor nodules (*P* = 0.032) and higher recurrence (*P* = 0.016). Over-expression of GPx3 or administration of rGPx3 significantly inhibited proliferation and invasiveness of HCC cells *in vitro* and *in vivo*. Tumor suppressive activity of GPx3 was mediated through Erk-NFκB-SIP1 pathway. GPx3 could be delivered by hiPSC-MSCs into the tumor and exhibited tumor suppressive activity *in vivo*.

Conclusions: GPx3 is a tumor suppressor gene in HCC and may possess prognostic and therapeutic value for HCC patients.

## INTRODUCTION

Oxidative stress is fatal to normal cells. In contrast, it plays a key role in cell survival and proliferation of several types of cancer [1]. Unlimited proliferation of cancer cells, to some extent, relies on intracellular oxidants such as reactive oxygen species (ROS). Oxidative stress could cause genetic mutation [2], up-regulate signaling pathways that control cell survival and invasiveness [3] and provide favorable micro-environment for cancer development [4]. Therefore, attenuation of oxidative stress post-operation or combined with chemotherapy may provide new insights for cancer treatment.

Glutathione peroxidase 3 (GPx3) was found to be up-regulated in acute phase injury as an anti-oxidant to protect the organ from oxidative stress by detoxifying hydrogen peroxide and other free radicals [5, 6]. It has also been noticed that expression of GPx3 is significantly down-regulated within tumor tissues in several types of cancers. The mRNA level of GPx3 is down-regulated in esophageal squamous cell carcinoma due to epigenetic silence [7]. The down-regulation of GPx3 was also observed in Barrett's adenocarcinoma because of DNA methylation [8]. Much lower expression levels of GPx3 were observed in colorectal cancer compared with non-tumor tissues [9]. Besides the down-regulation of GPx3 within tumor tissues, the circulating GPx3 was also found to be much lower in the patients with glioblastoma than non-patients which implies that it may possess the prognostic value for cancer patients [10]. Although the down-regulation of GPx3 was shown in several malignancies, the clinical significance and functional role of GPx3 in cancer development have not been well illustrated.

Hepatocellular carcinoma (HCC) is the sixth most common cancer and ranks as high as third for cancer-related deaths worldwide [11]. Although tumor resection and liver transplantation are effective treatments for selected HCC patients, tumor recurrence remains a main concern [12–10]. Furthermore, surgical treatment is not applicable for patients with advanced tumor stages [10–10]. Development of a new approach to prevent tumor recurrence and improve prognosis is an urgent need for HCC patients. The role of anti-oxidant in treatment of HCC patients has not been illustrated. So far, there is no information about the role of GPx3 in HCC. Exploration of functional role of GPx3 in HCC could provide new evidences to apply anti-oxidant agents for cancer therapy.

Firstly, we examined the clinical significance of GPx3 in HCC patients and found that lower expression of GPx3 in tumors could predict the advanced tumor stage and higher probability of tumor recurrence. Secondly, we explored the tumor suppressive activity of GPx3 *in vitro* by administration of rGPx3 or forced expression of GPx3 in different liver cancer cell lines. Thirdly, we demonstrated the anti-tumor effect of GPx3 *in vivo* using ectopic and orthotopic liver cancer models. Fourthly, we found that the tumor suppressive activity of GPx3 was mediated by inhibition of EMT (Epithelial-Mesenchymal Transition) through Erk-NFκB-SIP1 signaling pathway. Finally, we explored the therapeutic value of GPx3 for HCC using hiPSC-MSCs as a delivery vehicle. This is the first study to suggest the prognostic and therapeutic value of GPx3 in HCC patients.

## RESULTS

### Expression of GPx3 was down-regulated within tumor tissues in HCC patients

**Figure 1:**
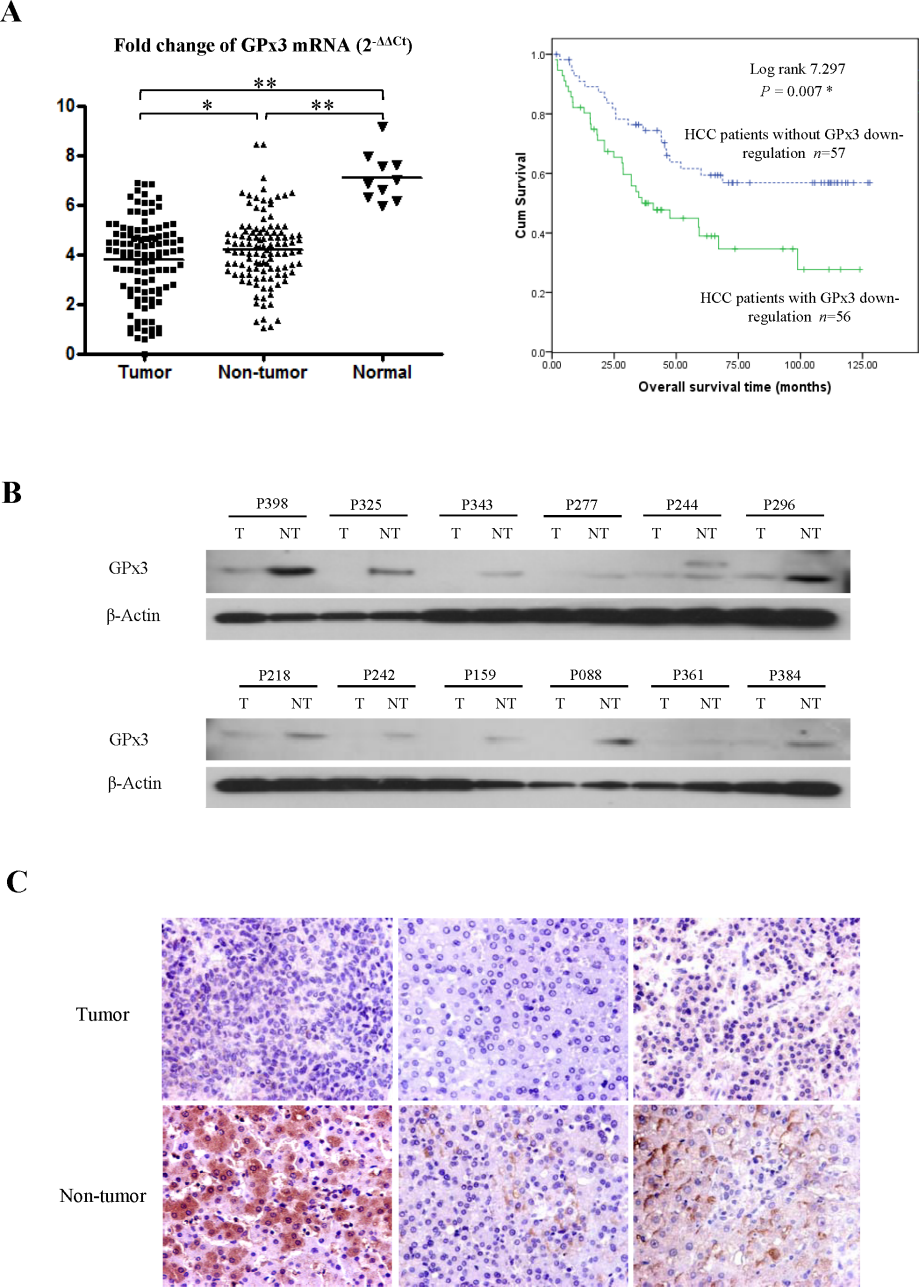
Down-regulation of GPx3 in HCC patients **(A)** GPx3 mRNA in clinical samples (Left panel), *P < 0.05, **P < 0.01. Overall survival of HCC patients underwent hepatectomy (Right penal), *P < 0.05. **(B)** Representative images of down-regulation of GPx3 in tumor tissues compared with adjacent non-tumor tissues by Western-Blot. **(C)** Representative images of HE staining showing the down-regulation of GPx3 within tumor tissue (400×).

The average mRNA level of GPx3 was significantly lower within tumor tissues compared with adjacent non-tumor tissues and normal liver tissues (Fig. 1A, left panel). The down-regulation of GPx3 in tumor tissues compared with adjacent non-tumor tissues was observed in 50% of HCC patients (56/113). It seemed that the difference between normal and diseased samples were larger than that between tumor and non-tumor tissues (Fig. 1A, left panel). There are two possible explanations. On one hand, HCC is regarded as inflammation associated cancer, which always develops with the background of cirrhosis. In such types of cancer, the chronic inflammation is always inevitable and would cause the lower level of GPx3 expression. On the other hand, the adjacent non-tumor tissue could be generally perceived as “pre-cancer” disease. Our results showed that GPx3 was already significantly down-regulated in such “pre-cancer” tissues. It implied that down-regulation of GPx3 may not only be involved in tumor progression, but also tumor development or carcinogenesis. Consistent with mRNA levels, the protein levels of GPx3 were also found down-regulated in tumor tissues (Fig. 1B). IHC staining also confirmed that lower expression of GPx3 was detected within tumor tissues (Fig. 1C).

### Down-regulation of GPx3 mRNA significantly correlated with advanced tumor stage and poor prognosis in HCC patients

The association of GPx3 mRNA with clinicopathological parameters was analyzed (Table 1). Down-regulation of GPx3 was significantly correlated with advanced pTNM stage (*P* = 0.024), presence of venous infiltration (*P* = 0.043) and high AFP level (*P* = 0.006). The five year recurrence (*P* = 0.019) was significantly higher in the patients with GPx3 down-regulation. No significant association of GPx3 expression was detected with sex, age, tumor size, number of tumor nodules and presence of hepatitis B surface antigen.

Kaplan-Meier survival analysis showed that the down-regulation of GPx3 mRNA was significantly correlated with poor overall survival of HCC patients (Log rank = 7.297, *P* = 0.007, Fig. 1A, right panel). The estimated mean overall survival time of the patients with GPx3 down-regulation was 58.72±6.94 months, which was significantly lower than that of patients without GPx3 down-regulation (86.46±6.85 months). The independent predictor for overall survival was further identified among GPx3, pTNM stage, venous infiltration and AFP using Cox proportional hazard regression analysis ( Table S1). In univariable analysis, down-regulation of GPx3 (HR=2.084, 95%CI: 1.209–3.593, *P* = 0.008) was significantly associated with overall survival. However, in multivariable analysis, only venous infiltration (HR=3.003, 95%CI: 1.259–7.160, *P* = 0.013) was identified as an independent predictor.

### Lower plasma GPx3 significantly correlated with tumor progression and tumor recurrence in HCC patients

The area under the ROC curve of plasma GPx3 was 0.643 (0.534–0.751) for prediction of five year recurrence. The value (4.842μg/mL) with a maximized Youden index was selected as the cutoff point by which all the patients were segregated into two groups: low and high level group. The larger tumor size (*P* = 0.011) and higher number of tumor nodules (*P* = 0.032) were found in the GPx3 low level group. The five year recurrence rate (*P* = 0.016) was significantly higher in the patients with low plasma GPx3 (Table 2). No significant association of plasma GPx3 was detected with age, sex, pTNM stage, venous infiltration and hepatitis B surface antigen. The patients with low plasma GPx3 had relatively shorter disease-free survival period (35.37±6.22 vs 49.14±7.11 months). However, the difference did not reach statistical significance (Log rank=2.044, *P* = 0.153).

**Table 1:** The correlation of GPx3 expression with clinical parameters of HCC patients

Clinicopathological features	GPx3 expression level (n)			
	Number	Down regulated	Non-down regulated	*P*
**Sex**				0.210
Male	92	43	49	
Female	21	13	8	
**Age**				0.926
Younger than 55	57	28	29	
Older than 55	56	28	28	
**pTNM stage^a^**				0.024 *
Early stage (1-2)	29	9	20	
Advanced stage (3-4)	83	46	37	
**Venous infiltration**				0.043 *
Absent	47	18	29	
Present	66	38	28	
**Tumor size^a^**				0.462
Less than 5 cm	47	25	22	
More than 5 cm	65	30	35	
**Tumor Nodules^a^**				0.448
Less than 3	83	39	44	
More than 3	29	16	13	
**HBsAg**				0.794
Negative	13	6	7	
Positive	100	50	50	
**AFP level^a^**				0.006 *
Lower than 20 ng/mL	45	15	30	
Higher than 20 ng/mL	67	40	27	
**One year recurrence^a^**				0.038*
Recurrence	42	27	15	
Non-recurrence	62	27	35	
**Five year recurrence^a^**				0.019*
Recurrence	62	36	26	
Non-recurrence	43	15	28	

a The total number less than 113 due to missing data.

* Significant difference.

**Table 2:** The correlation of plasma GPx3 with clinical parameters of HCC patients

Clinicopathological features	Plasma GPx3 level (n)			*P*
	Number	Low level^b^	High level^b^	
**Sex**				0.835
Male	88	44	44	
Female	19	9	10	
**Age**				0.210
Younger than 55	52	29	23	
Older than 55	55	24	31	
**pTNM stage^a^**				0.353
Early stage (1-2)	24	10	14	
Advanced stage (3-4)	82	43	39	
**Venous infiltration**				0.750
Absent	42	20	22	
Present	65	33	32	
**Tumor size^a^**				0.011*
Less than 5 cm	45	16	29	
More than 5 cm	61	37	24	
**Tumor Nodules^a^**				0.032*
Less than 3	77	34	43	
More than 3	28	19	9	
**HBsAg**				0.518
Negative	12	7	5	
Positive	95	46	49	
**AFP level^a^**				0.691
Lower than 20 ng/mL	42	22	20	
Higher than 20 ng/mL	64	31	33	
**One year recurrence^a^**				0.227
Recurrence	42	24	18	
Non-recurrence	60	27	33	
**Five year recurrence^a^**				0.016*
Recurrence	61	36	25	
Non-recurrence	38	13	25	

a The total number less than 107 due to missing data.

b The cutoff point with maximized Youden index was 4.842μg/mL by which all the patients were segregated into two groups.

* Significant difference.

### Expression level of GPx3 in normal and HCC cell lines

**Figure 2:**
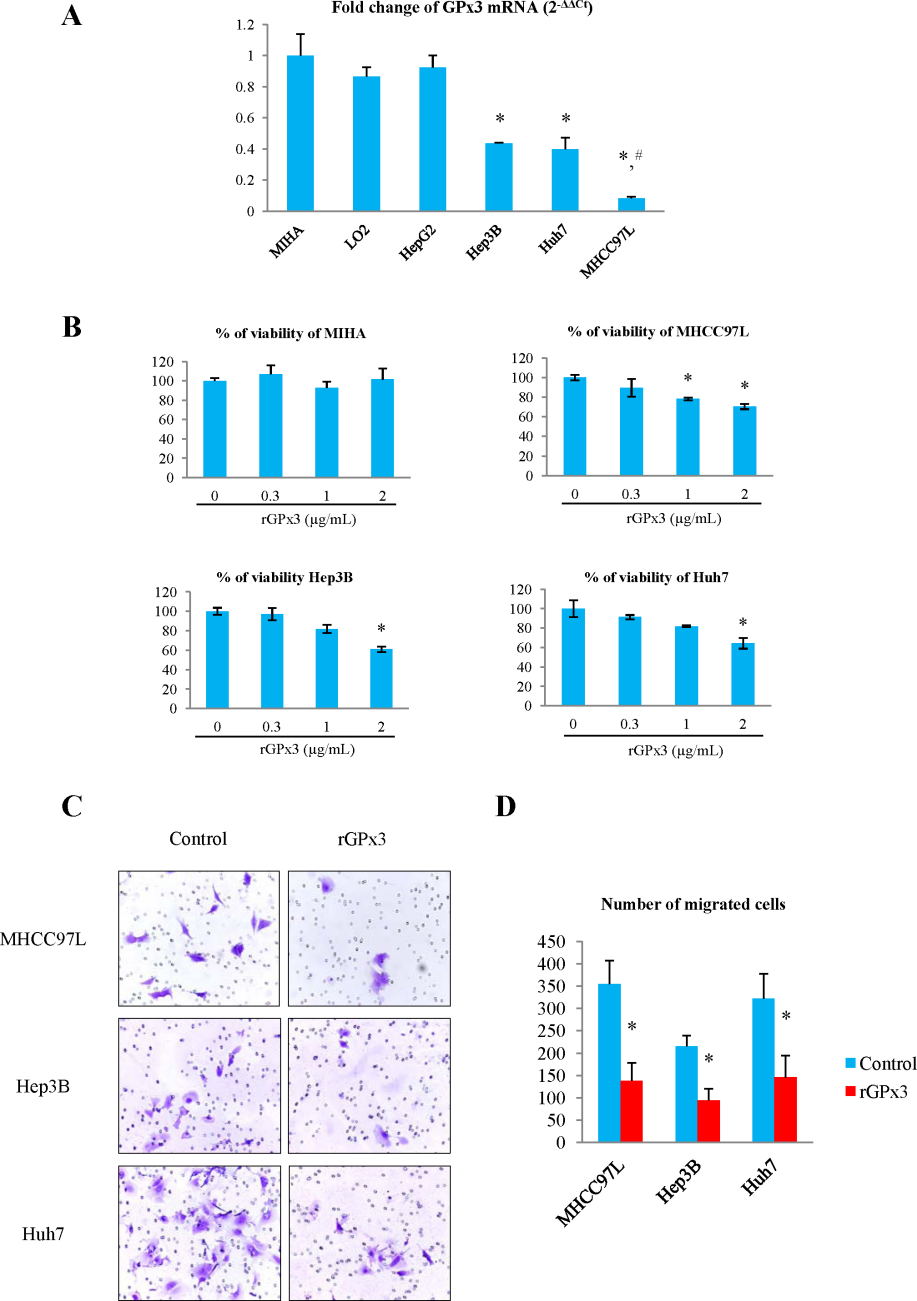
Effects of rGPx3 on HCC cells **(A)** GPx3 mRNA in different cell lines, *P < 0.05 *vs* MIHA and LO_2_; ^#^ *P* < 0.05 *vs* Hep3B and Huh7. **(B)** The effect of rGPx3 on proliferation of different cells, *P < 0.05 *vs* 0 μg/mL. **(C** and **D)**: The effect of rGPx3 on invasiveness of HCC cells.

The mRNA level of GPx3 was significantly lower in HCC cell lines (except HepG2) compared with immortalized liver cell lines (MIHA and LO_2_) (Fig. 2A). Among HCC cell lines, the expression of GPx3 was significantly lower in metastatic HCC cell line (MHCC97L) compared with non-metastatic HCC cell lines (Hep3B and Huh7).

### Recombinant GPx3 protein (rGPx3) or over-expression of GPx3 inhibited proliferation and invasiveness of HCC cells *in vitro*

Administration of rGPx3 inhibited the proliferation of MHCC97L, Hep3B and Huh7, whose GPx3 was relatively low (Fig. 2B). The proliferation of HCC cells was most significantly suppressed by rGPx3 at the concentration of 2μg/mL, which was shown to be non-toxic to normal liver cells (MIHA) (Fig. 2B). The invasiveness of MHCC97L, Hep3B and Huh7 was also suppressed by administration of rGPx3 (Fig. 2C). The number of migrated cells was significantly lower upon rGPx3 administration in the Matrigel invasion assay (Fig. 2D).

**Figure 3:**
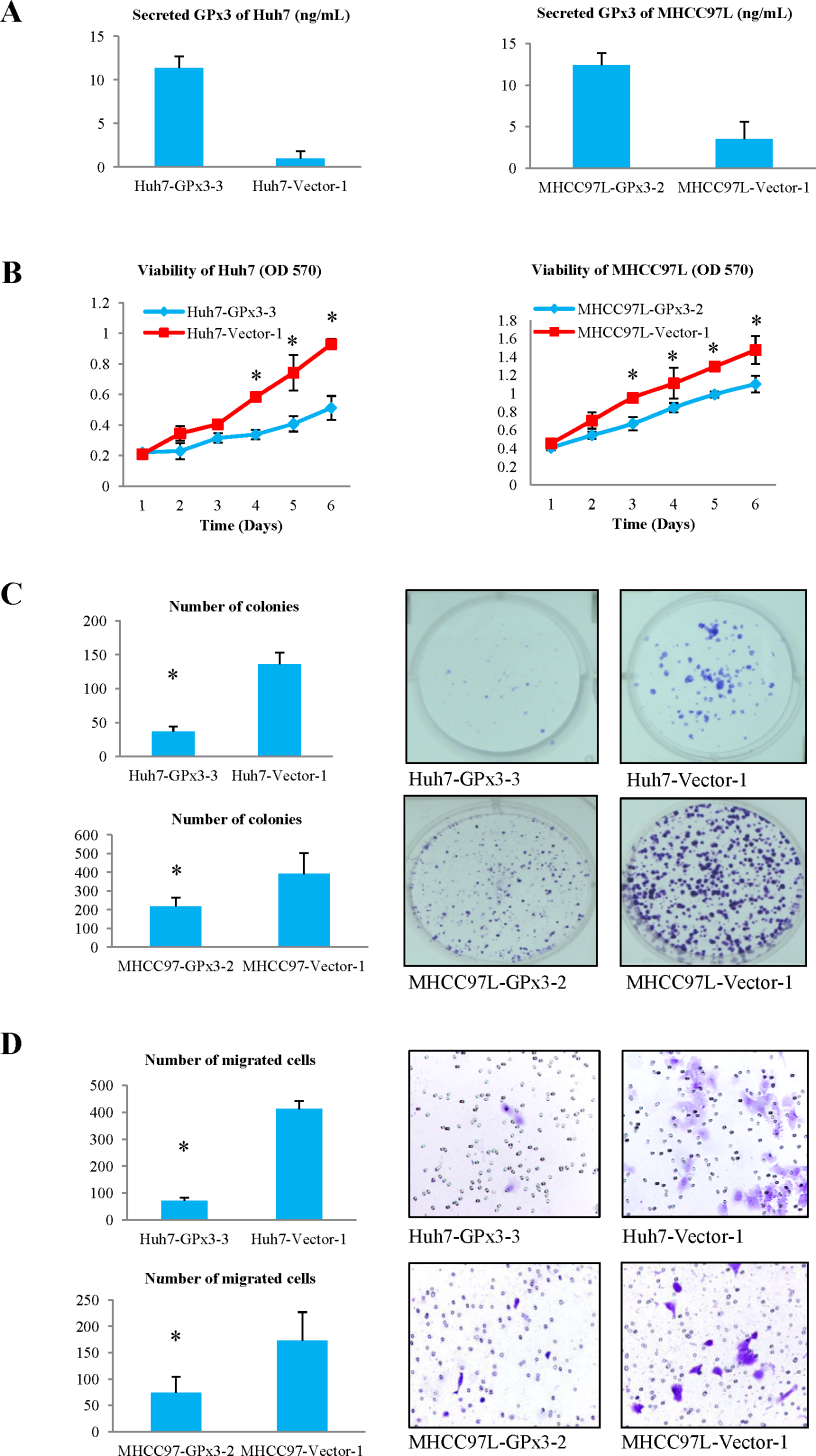
Effects of over-expression of GPx3 on HCC cells **(A)** Secreted GPx3 in culture medium. **(B)** MTT assay, *P < 0.05. **(C)** Colony formation assay, *P < 0.05. **(D)** Matrigel invasion assay, *P < 0.05.

Over-expression of GPx3 was established in Huh7 and MHCC97L. The clones with the highest expression of GPx3 were picked up for further investigation (Fig. S1A and B). We also confirmed that secreted GPx3 was significantly increased in the culture medium after transfection (Fig. 3A). It implied that the forcedly expressed GPx3 could be successfully secreted extracellularly. Over-expression of GPx3 significantly inhibited the proliferation (Fig. 3B) of Huh7 and MHCC97L in MTT assay. Moreover, in colony formation assay, the colony number and size were both significantly lower in GPx3 over-expression group (Fig. 3C). Furthermore, the invasiveness was also significantly suppressed by over-expression of GPx3 in Huh7 and MHCC97L (Fig. 3D).

### Over-expression of GPx3 suppressed tumor growth and invasiveness *in vivo*

**Figure 4:**
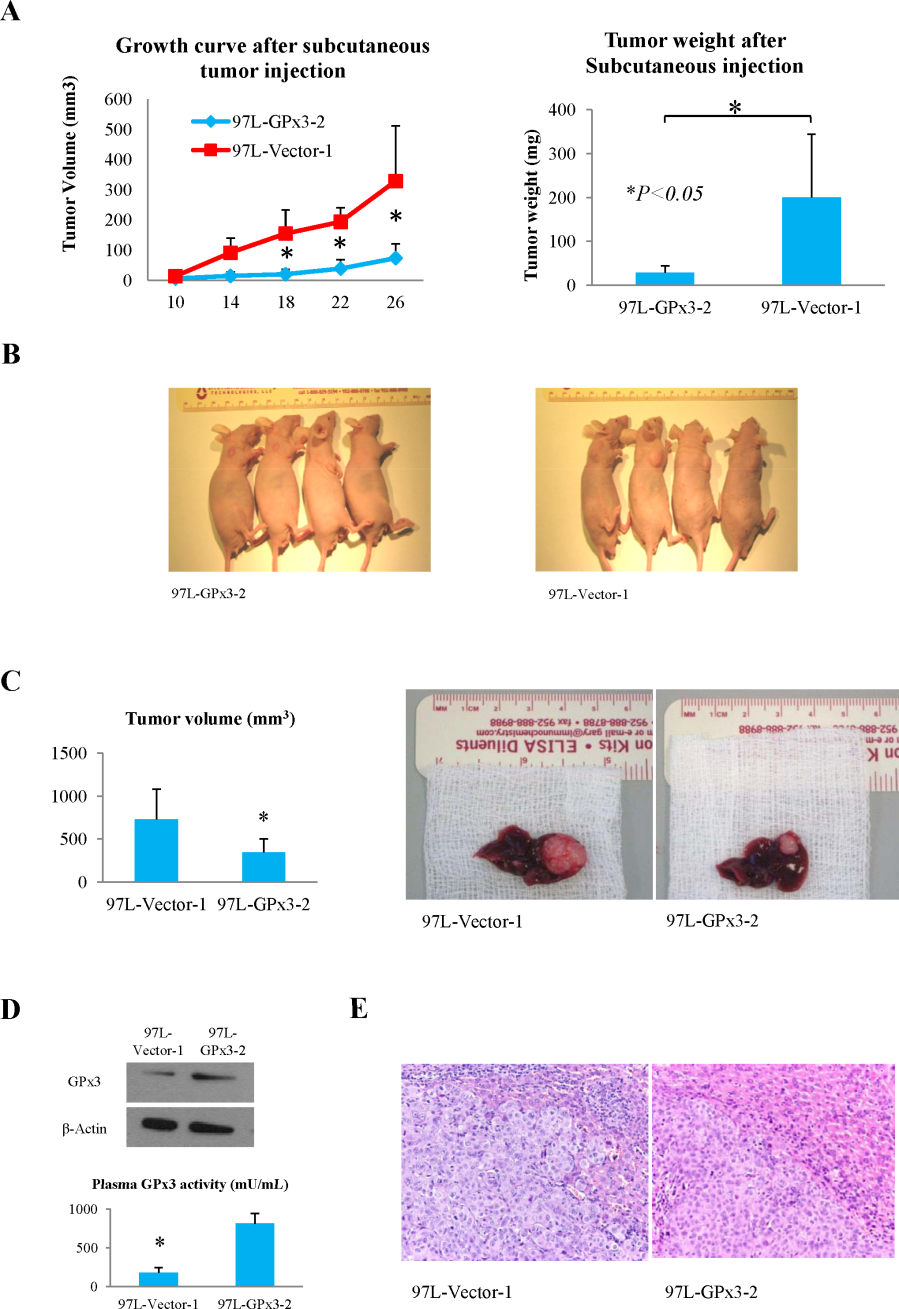
Effects of over-expression of GPx3 on tumor growth in nude mice ectopic and orthotopic liver cancer model **(A** and **B)** Over-expression of GPx3 significantly inhibited subcutaneous tumor growth, *P < 0.05. **(C)** Over-expression of GPx3 significantly inhibited orthotopic tumor growth, *P < 0.05. **(D)** Over-expression of GPx3 could be maintained 5 weeks after implantation. **(E)** Representative images of HE staining (400×).

In nude mice ectopic liver cancer model, the tumor growth rate of MHCC97L-GPx3-2 was significantly lower compared with vector control (Fig. 4A and B). In nude mice orthotopic liver cancer model, tumor volume was significantly lower in MHCC97L-GPx3-2 group (Fig. 4C). The over-expression of GPx3 in MHCC97L-GPx3-2 group was also confirmed 5 weeks after tumor implantation (Fig. 4D). It implied that forced-expression of GPx3 could be sustained *in vivo* and secreted extracellularly. A relatively clear margin between tumor and adjacent non-tumor tissues was found in the MHCC97L-GPx3-2 group. On the contrary, invasive patterns of tumor growth were present in vector control group (Fig. 4E). The results showed that over-expression of GPx3 suppressed tumor invasiveness *in vivo*.

### GPx3 inhibited Epithelial-Mesenchymal Transition (EMT) through suppression of Smad Interacting Protein 1 (SIP1)

**Figure 5:**
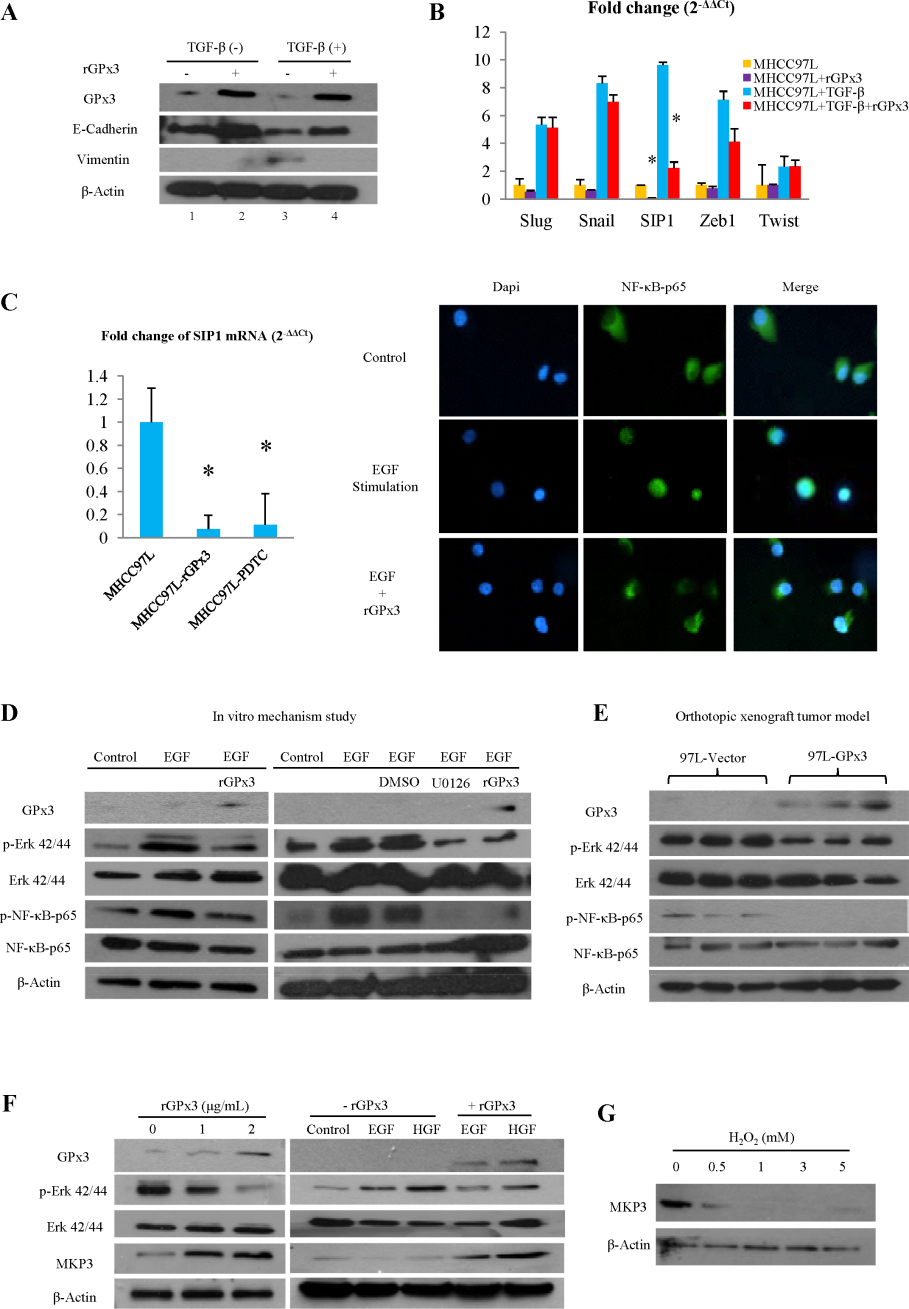
Potential mechanism for tumor suppressive activity of GPx3 **(A)** GPx3 inhibited EMT in MHCC97L. **(B)** GPx3 inhibited EMT through suppression of SIP1. **(C)** GPx3 suppressed expression of SIP1 through inhibition of NFκB nuclear translocation (right panel). SIP1 expression could be regulated by NFκB pathway evidenced by effect of specific NFκB inhibitor (PDTC, left panel). **(D)** GPx3 inhibited activation of NFκB through deactivation of Erk (left panel). Activation of NFκB could be regulated by Erk pathway evidenced by effect of specific Erk inhibitor (U0126, right panel). **(E)** Deactivation of NFκB and Erk by GPx3 was confirmed in orthotopic tumor model. **(F)** GPx3 inhibited Erk activation in a dose dependent manner and attenuated EGF- and HGF- induced Erk activation through restoration of MKP3 expression. **(G)** Expression of MKP3 in MHCC97L was negatively regulated by H_2_O_2_.

As significant anti-invasiveness effect of GPx3 was observed *in vitro* and *in vivo* (Fig. 2C, 3D and 4E), we further investigated the effect of GPx3 on EMT process. GPx3 significantly inhibited EMT which was stimulated by TGF-β in MHCC97L (Fig. 5A). GPx3 restored the expression of E-cadherin (epithelial marker) and decreased expression of Vimentin (mesenchymal marker) of MHCC97L.

In order to further investigate the underlying mechanism of inhibitory effect of GPx3 on EMT, several transcriptional repressors of E-cadherin that are commonly involved in EMT process, such as Slug, Snail, Zeb1, SIP1 and Twist, were selected for detection. In our case, SIP1 was shown to be most significantly suppressed by GPx3 treatment independent of TGF-β stimulation (Fig. 5B).

### GPx3 suppressed SIP1 expression through inhibition of Nuclear Factor κB (NFκB) activation

NFκB was reported to be directly involved in EMT process and is the key mediator for SIP1 expression [19]. In our study, SIP1 expression could be regulated by NFκB pathway as administration of specific NFκB inhibitor (PDTC) suppressed SIP1 expression (Fig. 5C, left panel). Furthermore, NFκB nuclear translocation was significantly attenuated upon rGPx3 administration, indicating that activation of NFκB could be inhibited by GPx3 treatment (Fig. 5C, right panel). Thus, GPx3 treatment might inhibit NFκB activation, which would further suppress SIP1 expression.

### GPx3 inhibited NFκB activation through deactivation of Extracellular signal-regulated kinase (Erk)

PI3K-AKT and Ras-MEK-Erk signaling pathways are both commonly involved in liver cancer progression and are reported to be the activators of the NFκB signaling pathway [20, 21]. In our case, the activated form of AKT remained unchanged upon rGPx3 administration (Fig. S2A). However, significant inhibition of Erk activation was observed upon rGPx3 administration (Fig. 5D, left panel). Furthermore, activation of NFκB could be regulated by the Erk pathway as evidenced by how the administration of specific Erk inhibitor (U0126) deactivated NFκB-p65 (Fig. 5D, right panel). Thus, GPx3 might inhibit Erk activation, which would further deactivate NFκB signaling pathway. The same trend could also be observed in orthotopic liver cancer model that activation of NFκB was inhibited by the over-expression of GPx3 through deactivation of Erk (Fig. 5E).

### GPx3 deactivated Erk by up-regulation of MAP Kinase Phosphatase 3 (MKP3)

We further observed that the activation of Erk could be inhibited by GPx3 in a dose dependent manner (Fig. 5F, left panel). However, the up-stream stimulators of the Erk signaling pathway, such as Raf and MEK, were not changed upon GPx3 treatment (Fig. S2A). It implied that deactivation of Erk by GPx3 may not be mediated through Raf-MEK pathway. It was reported that the negative feedback of Erk activation was regulated by MKP3 [22], which was Erk phosphatase and widely expressed in liver tissue. In our case, MKP3 was significantly up-regulated upon rGPx3 treatment in a dose dependent manner (Fig. 5F, left panel). Furthermore, GPx3 could attenuate EGF (Epidermal Growth Factor) and HGF (Hepatocyte Growth Factor) induced Erk activation through the restoration of MKP3 expression (Fig. 5F, right panel). Thus, the inhibitory effect of GPx3 on Erk activation might be mediated through up-regulation of MKP3, which could maintain the negative feedback loop of Erk activation (Fig. S3). Moreover, our study showed that expression of MKP3 could be negatively regulated by H_2_O_2_ level (Fig. 5G). Thus, the restoration of MKP3 expression by GPx3 might be mediated through elimination of H_2_O_2_ due to the detoxification of GPx3.

### Engineered hiPSC-MSCs delivering GPx3 was established

In order to explore the therapeutic value of GPx3 using hiPSC-MSCs as a delivery vehicle in HCC, we established the engineered hiPSC-MSCs delivering GPx3. Full length of human GPx3 gene was transduced into the hiPSC-MSC cell line [23]. The transduction efficiency was confirmed to be nearly 100% (Fig. S4A). The protein level of intracellular GPx3 and secreted GPx3 in culture medium were both confirmed to be significantly elevated after transduction (Fig. S4B). The stem cell property of hiPSC-MSCs was still maintained after transduction as evidenced by positive and negative markers detected using flow cytometry (Fig. S5).

### Engineered hiPSC-MSCs delivering GPx3 significantly suppressed tumor growth *in vitro*

**Figure 6:**
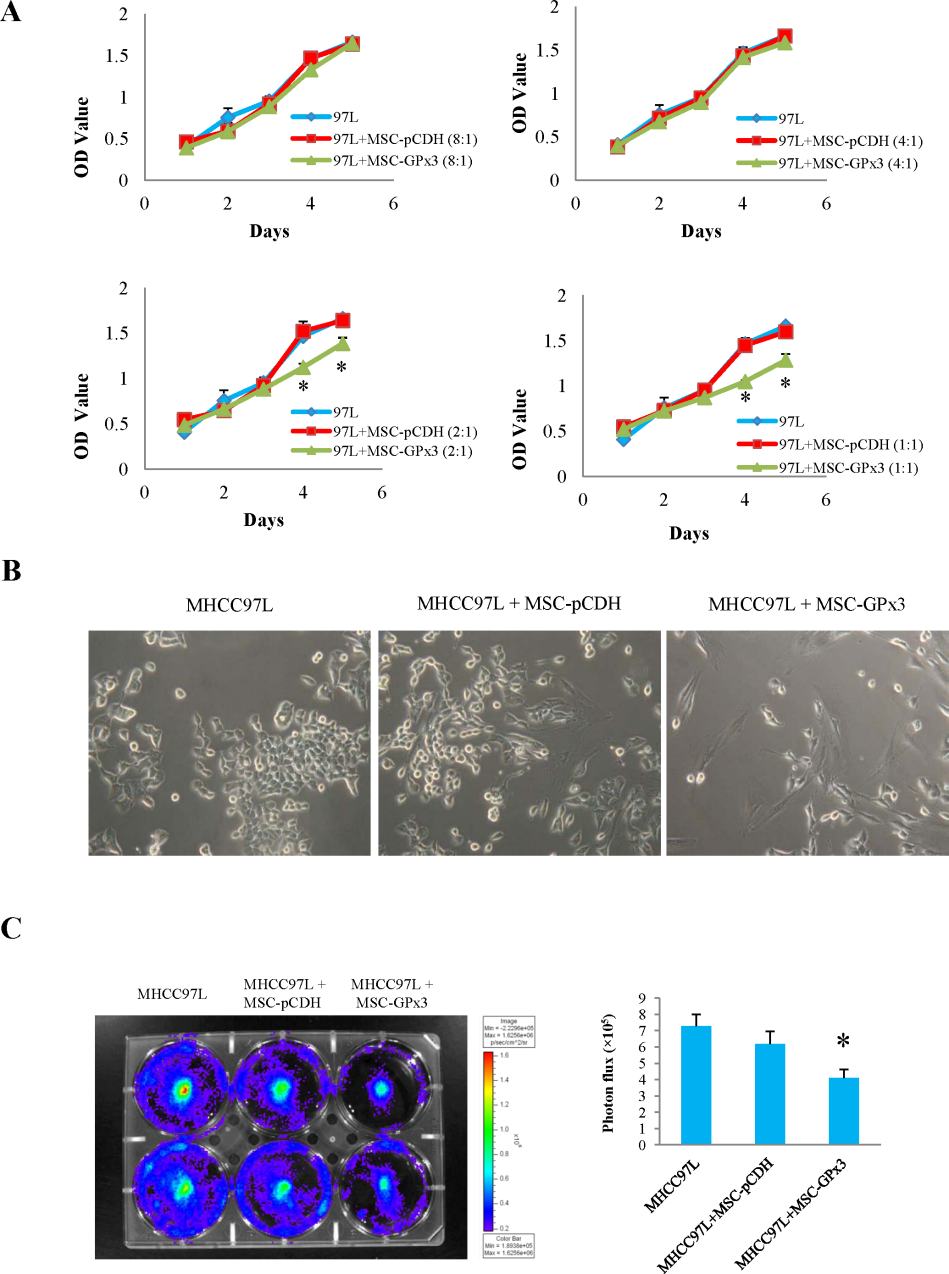
Tumor suppressive activity of hiPSC-MSC-GPx3 *in vitro* **(A)** hiPSC-MSC-GPx3 significantly inhibited proliferation of MHCC97L in MTT assay, *P<0.05. **(B** and **C)** hiPSC-MSC-GPx3 significantly inhibited proliferation of MHCC97L shown by bioluminescence, *P<0.05.

The significant inhibitory effect of hiPSC-MSC-GPx3 on the proliferation of MHCC97L cells was observed when the cell number ratio reached 2:1 or 1:1 (MHCC97L:hiPSC-MSC-GPx3) in MTT assay (Fig. 6A). In order to avoid the influence of proliferation of hiPSC-MSCs in detection, hiPSC-MSC-GPx3 was co-cultured with MHCC97L cells labeled with luciferase (Fig. 6B). The bioluminescence of MHCC97L was significantly lower in the hiPSC-MSC-GPx3 group. It implied that hiPSC-MSC-GPx3 could significantly suppress the proliferation of MHCC97L cells (Fig. 6C).

### Engineered hiPSC-MSCs delivering GPx3 significantly suppressed tumor growth *in vivo*

**Figure 7:**
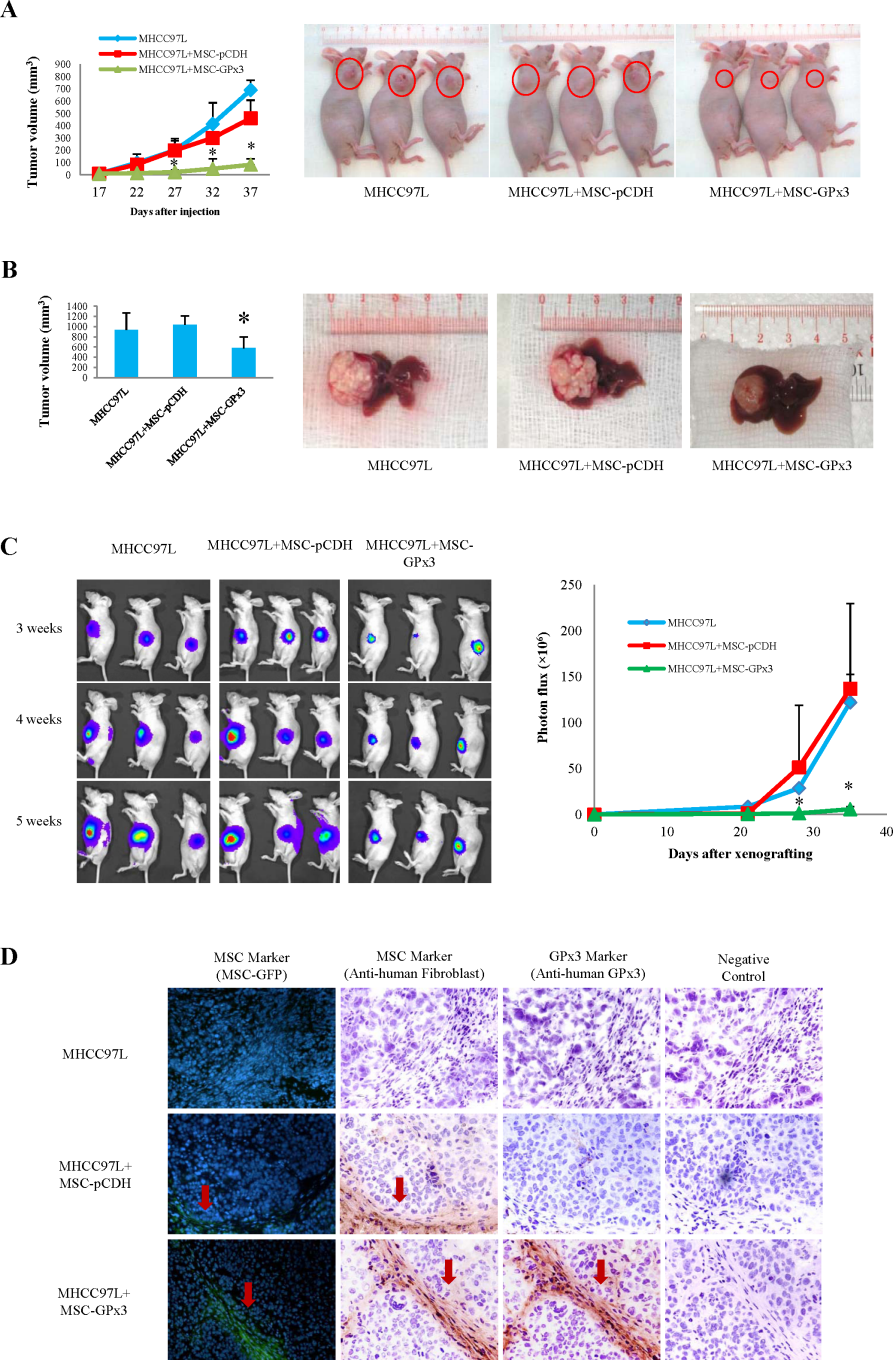
Tumor suppressive activity of hiPSC-MSC-GPx3 *in vivo* **(A)** Subcutaneous tumor growth curve was significantly lower in hiPSC-MSC-GPx3 group. **(B)** Orthotopic tumor volume was significantly lower in hiPSC-MSC-GPx3 group. **(C)** Kinetic images of tumor growth showed tumor suppressive activity of hiPSC-MSC-GPx3, *P<0.05. **(D)** Successful delivery of GPx3 by hiPSC-MSCs in tumor tissues. Pictures in each group showed the same area of tissue sections (400×).

The subcutaneous tumor growth curve was significantly lower in the hiPSC-MSC-GPx3 treatment group (Fig. 7A). In the orthotopic model, the tumor volume was significantly lower in the hiPSC-MSC-GPx3 treatment group when nude mice were sacrificed (Fig. 7B). The kinetic images of tumor growth also showed that hiPSC-MSC-GPx3 treatment significantly suppressed orthotopic tumor growth *in vivo* (Fig. 7C). According to immunoflorescence and IHC staining, human hiPSC-MSCs could be traced within tumor tissues (Fig. 7D, middle panel). Moreover, GPx3 expression could be detected at the same area at which hiPSC-MSC-GPx3 cells were located (Fig. 7D, lower panel). It implied that GPx3 could be successfully delivered by hiPSC-MSCs into tumor tissues.

## DISCUSSION

We demonstrated that the lower plasma GPx3 indicated tumor recurrence and shorter disease-free-survival period of HCC patients after liver resection. The down-regulation of GPx3 within tumor tissue also significantly correlated with advanced tumor stage and appearance of venous infiltration in HCC patients. These findings implied that GPx3 may possess prognostic value to predict HCC progression and recurrence. Although numerous biomarkers have been studied in past decades, the serological detection of HCC recurrence mainly relies on the traditional marker AFP [4, 24]. However, the positive predictive value (PPV) of AFP is not satisfied [25]. Furthermore, as the secreted proteins originated from tumors, AFP does not possess therapeutic value for HCC patients. So far, there is no promising biomarker possessing both prognostic and therapeutic value for HCC patients. In addition to indicating the clinical significance of GPx3, we also illustrated that over-expression of GPx3 in HCC cells significantly suppressed tumor proliferation and invasiveness *in vitro* and *in vivo*. More importantly, we demonstrated, for the first time that the tumor suppressive activity of GPx3 delivered by hiPSC-MSCs indicated the therapeutic potential of hiPSC-MSC-GPx3 for HCC patients. Based on the advantages that patient specific hiPSC-MSCs could be prepared as an “off-the-shelf” source with a higher capacity of self-renewal and engraftment [23], the clinical application of hiPSC-MSC-GPx3 may be a practical choice to prevent tumor recurrence and improve prognosis of HCC patients after liver surgery.

Epigenetic hypermethylation and genome deletion are reported as two reasons for the down-regulation of GPx3 during cancer development [7, 8, 26, 27], but the mechanism of down-regulation of GPx3 contributing to tumor progression remains to be investigated. GPx3 has been reported to commit its tumor suppressive activity through inhibition of c-Met expression in prostate cancer [28]. However, we did not find any difference of c-Met expression in HCC cell lines with forced expression of GPx3 (Fig. S2B). It indicated that tumor suppressive activity of GPx3 through down-regulation of c-Met may be cell-type-specific. Moreover, several reports demonstrated that the tumor suppressive function of GPx3 was mediated through induction of apoptosis in prostate and colon cancer [29, 30]. However, in our study, no significant effect of GPx3 on apoptosis of HCC cells was observed (Fig. S1C). Consistently, we did not find any change in cell survival pathway, such as AKT pathway, after over-expression of GPx3 (Fig. S2A). It implied that other underlying mechanism might be existed for tumor suppressive activity of GPx3 in liver cancer. This is the first study to show that rGPx3 suppressed invasiveness of HCC cells by inhibition of EMT through Erk-NFκB-SIP1 signaling pathway. It has been reported that activation of NFκB could be determined by the Erk pathway [31]. It was consistent with our results. In addition, the regulatory effect of NFκB on SIP1 expression is also supported by Julien, who demonstrated that activation of NFκB promotes SIP1 expression which further suppresses E-cadherin expression [32]. We identified that only inhibition of SIP1, among five E-cadherin transcriptional repressors, was responsible for restoration of E-cadherin by rGPx3. So far, there is no promising SIP1 inhibitor for cancer therapy except for epigenetic silencing [33], in which the negative side effects may be inevitable. Besides that, the application of inhibitors of the NFκB pathway for cancer therapy is still at an early stage because of several severe side effects [34]. The hiPSC-MSC-GPx3 treatment with property of tumor tropism may be prospective for clinical utilization of cancer target therapy in HCC patients.

In addition to anti-invasiveness, significantly inhibitory effect of GPx3 or hiPSC-MSC-GPx3 on proliferation of HCC cells was also observed *in vitro* and *in vivo*. We found that GPx3 could maintain negative feedback of Erk activation by up-regulation of MKP3, which further deactivated the NFκB pathway. The inhibitory effect of GPx3 on Erk-NFκB activation might be able to explain the suppressive effect of GPx3 on both proliferation and invasiveness of HCC cells. This was confirmed in our animal model (Fig. 5E). Further research is required to understand whether there are other potential mechanisms involved in the tumor suppressive activity of GPx3.

Engineered MSC with an anti-tumor agent was reported as a novel strategy for cancer therapy with the advantage of fewer systemic side effects [35, 36]. However, the self-renewal capacity of bone-marrow-derived-MSCs (BM-MSCs) was significantly deteriorated with aging [37]. The hiPSC-MSCs were discovered to possess a higher capacity of self-renewal with telomerase activity 10-fold higher than BM-MSCs [23]. Thus, it was of our great interest to explore the therapeutic value of GPx3 using hiPSC-MSCs as a delivery vehicle for HCC. The co-localization of hiPSC-MSCs and GPx3 expressions detected within tumor tissues highly implied that GPx3 can be successfully delivered by hiPSC-MSCs into the tumor site. This finding might provide a new delivery strategy for GPx3 in liver cancer target therapy.

We suggested that the lower circulating GPx3 possessed prognostic value for HCC patients. We not only investigated the clinical significance, but also further demonstrated the tumor suppressive role of GPx3 in HCC *in vitro* and *in vivo*. Moreover, we discovered the potential mechanism of GPx3 that suppressed invasiveness of tumor by inhibition of EMT through the Erk-NFκB-SIP1 signaling pathway. Most importantly, we further demonstrated therapeutic value of GPx3 using hiPSC-MSCs as a delivery vehicle in liver cancer models. Further investigation is required to explore the therapeutic effect of hiPSC-MSC-GPx3 combined with other anti-tumor agents.

## MATERIALS AND METHODS

### Study design

The flow diagram of study design is shown in Fig. S6. In clinical association study, the correlation of GPx3 expression with clinicopathological features and survival outcome were investigated in 113 HCC patients, who had undergone hepatectomy. In functional study, the effects of GPx3 on HCC cells were examined by administration of rGPx3 and forced expression of GPx3 in HCC cells *in vitro*, and the *in vivo* role of GPx3 was explored in ectopic and orthotopic xenograft liver cancer models. In mechanism study, effects of GPx3 on the EMT process and the activation of Erk-NFκB-SIP1 pathway were further explored. In translational study, the therapeutic value of GPx3 in HCC was explored using hiPSC-MSCs as a delivery vehicle *in vitro* and *in vivo*.

### Clinical association study

#### Clinical specimens

The study was approved by the Institutional Review Board of the University of Hong Kong / Hospital Authority Hong Kong West Cluster. 113 HCC patients who had undergone curative partial hepatectomy between November 1999 and May 2009 were recruited with informed consent from the Department of Surgery, Queen Mary Hospital, The University of Hong Kong. All the patients were diagnosed with primary HCC and the patients with severe systemic disorder or combined with other tumors were excluded from the study. The median of follow up time was 6 years (2 months – 11 years). The median age of the patients was 55 (22–81) years old. Eighty one percent of patients were male. Eighty eight percent of patients were HBsAg positive. One hundred and thirteen pairs of fresh tumor tissue and adjacent non-tumor tissue samples were collected from HCC patients and 10 normal liver tissue samples were collected from donors during operation. One hundred and seven plasma samples were collected within a week pre-operation.

#### Real time quantitative reverse transcription polymerize chain reaction (qRT-RCR), Immunohistochemistry (IHC) and Western-Blot

The mRNA level of GPx3 was determined by qRT-PCR. The protein level of GPx3 was detected by IHC and Western-blot as previously described [38]. GPx3 primary anti-body (Abcam) was used in immunostaining. The sequences of primers used were recorded ( Table S2).

#### Enzyme-linked immunosorbent assay (ELISA)

The plasma samples were diluted 800 times and the medium for cell culture was without any dilution before detection. The concentration of GPx3 was tested using ELISA kit according to instruction manual (AdipoGen Inc, Incheon, Korea).

### *In vitro* functional study

#### Cell culture, transfection and stable cell lines

Human liver cancer cell lines (HepG2, Hep3B and Huh7) and human immortal liver cell lines (MIHA and LO_2_) were purchased from the American Type Culture Collection (Manassas, VA, USA). The metastatic human liver cancer cell line MHCC97L was obtained from the Liver Cancer institute and Zhongshan Hospital of Fudan University, Shanghai, the People's Republic of China [39]. All the cell lines were cultured as previously described [40]. The pcDNA 3.1 (+) vector was purchased from Invitrogen (Carlsbad, CA). MHCC97L and Huh7 cells were transfected with recombinant plasmid using lipofectamine 2000 Reagent (Invitrogen, Carlsbad, CA) and cultured for two weeks under G418 selection. Stable clones were maintained in 0.05 mg/mL G418 for Huh7 cells and 0.5 mg/mL for MHCC97L cells, respectively.

#### 3-(4,5-dimethylthiazol-2-yl)-2,5-diphenyltetrazolium bromide (MTT) assay and Colony formation assay

In order to explore the proliferation rate of HCC cells, MTT and colony formation assay were performed as previously described [40].

#### Matrigel invasion assay

Around 5×10^4^ cells were seeded into the upper chamber (BD Biosciences) in 0.5 ml of serum-free DMEM. In the meantime, the lower chamber was filled up using DMEM with 10% FBS as a chemoattractant. The cells were incubated at 37°C with 5% CO_2_ for 24h for Huh7 and 48h for MHCC97L, respectively. After that, cells left on the upper surface of the chamber were removed gently using a cotton tip. Cells that had penetrated through the membrane of chamber were fixed by methanol at −20ºC for 20min, stained with 0.1% crystal violet for 1h and finally counted under a light microscope. The experiment was performed in triplicate.

### *In vivo* functional study

#### Nude mice ectopic tumorigenesis model

MHCC97L cells (1×10^6^ cells in 100ul saline) were injected subcutaneously into the right flank of the nude mice (6-8weeks, male) under anaesthesia with an intraperitoneal injection of pentobarbital. When the tumor developed, the size was measured every 4 days for 3 weeks. The volume of the tumor was calculated as follows: tumor volume (mm^3^) = 1/2 × length × width^2^. Tumor weight was measured when the mice were sacrificed at 4 weeks after the injection of tumor cells. Tumor tissues were harvested for further analysis. Six mice were recruited for each of the experimental group. The study had been licensed according to Animal (Control of Experiments) Ordinance Chapter 340 by the Department of Health, Hong Kong Special Administrative Region. (ref.: (11–632) in DH/HA&P/8/2/3 Pt. 31).

#### Nude mice orthotopic xenograft liver cancer model

When the subcutaneous tumors grew and reached 6mm × 6mm in size, the animals were sacrificed by an overdose intraperitoneal injection of pentobarbital. The tumor tissues were harvested and cut into 1-2mm^3^ cubes. The tumor tissue cubes were then implanted into the left liver lobes of another group of mice under anaesthesia as described previously [41]. Five weeks after the tumor implantation, the animals were sacrificed for samples collection and further analysis. Eight mice were recruited in each group. The study had been licensed according to Animal (Control of Experiments) Ordinance Chapter 340 by the Department of Health, Hong Kong Special Administrative Region. (ref.: (11–632) in DH/HA&P/8/2/3 Pt. 31).

### Mechanism study

#### EMT detection

In order to explore the mechanism of anti-invasiveness of rGPx3, the role of rGPx3 in the EMT process was detected. EMT of MHCC97L was pre-induced by TGF-β *in vitro* and then treated with rGPx3. The epithelial and mesenchymal markers were detected afterwards. Five E-cadherin transcriptional repressors, Slug, Snail, SIP1, Zeb1 and Twist, which were commonly involved in the EMT process, were detected to identify which one was responsible for the inhibitory effect of rGPx3 on EMT. Details were listed in supplementary material.

#### Regulation of NFκB signaling pathway

In order to explore whether the suppressive effect of rGPx3 on SIP1 was through inhibition of the NFκB signaling pathway, the activation of NFκB in MHCC97L was pre-induced by EGF and then treated with rGPx3. The nuclear translocation of NFκB was observed by immunoflorescence. To explore whether the down-regulation of SIP1 could be directly regulated by the NFκB signaling pathway, NFκB inhibitor (pyrrolidine dithiocarbamate, PDTC, Sigma) was used. Details were listed in supplementary material.

##### Regulation of Erk signaling pathway

In order to investigate whether the inhibitory effect of rGPx3 on NFκB was through inhibition of Erk pathway, the activation of Erk was pre-induced in MHCC97L by EGF *in vitro* and then treated with rGPx3. To explore whether activation of NFκB could be directly regulated by Erk signaling pathway, Erk inhibitor (U0126) was used. Details were listed in supplementary material.

##### Regulation of MKP3 expression

In order to explore whether inhibitory effect of rGPx3 on Erk activation was through regulation of MKP3, expression of MKP3 were detected in MHCC97L after rGPx3 administration. To explore whether MKP3 expression was regulated by ROS levels, different concentrations of H_2_O_2_ administration were applied. Details were listed in supplementary material.

#### Translational study

##### Establishment of engineered hiPSC-MSCs delivering GPx3

A full-length human GPx3 gene was transduced into expression plasmid pCDH-CMV-MCS-EF1-copGFP (System Biosciences, SBI). Virus particles were packaged in 293TN cells (System Biosciences, SBI). The hiPSC-MSCs cell line was derived from our collaborator Dr. Lian [23]. Expression of GPx3 after transduction was confirmed by western-blot and Elisa. Stem cells' property was investigated after transduction using flow cytometry. Antibodies of positive markers CD44-PE, CD105-PE, CD90-PE and CD73-PE and negative markers CD34-PE and CD45-PE were purchased from Biosciences (BD).

##### Investigation of tumor suppressive role of hiPSC-MSC-GPx3 *in vitro*

The effect of hiPSC-MSC-GPx3 on the proliferation of MHCC97L cells was examined by MTT assay. MHCC97L was co-cultured with hiPSC-MSC-GPx3 at a different ratio for different time period. As hiPSC-MSC-GPx3 could hardly proliferate in the medium of MHCC97L, the OD value of MTT assay mainly reflects the proliferation of MHCC97L cells. In order to distinguish two types of cells in co-culture system, MHCC97L (1×10^4^/well) was labeled with luciferin and co-cultured with hiPSC-MSC-GPx3 (5×10^3^/well). Proliferation of MHCC97L in co-culture system was shown as luciferin signal detected by Xenogen IVIS^®^ *in vivo* imaging system.

##### Investigation of tumor suppressive role of hiPSC-MSC-GPx3 *in vivo*

The nude mice ectopic and orthotopic xenograft liver cancer models were established as described in section of *in vivo* functional study above. In the ectopic xenograft liver cancer model, MHCC97L (2×10^6^/100μL) was co-injected with hiPSC-MSC-GPx3 (1×10^6^/100μL) subcutaneously into the right flank of nude mice. Tumor size was measured every 5 days for 3 weeks. In orthotopic xenograft liver cancer model, hiPSC-MSC-GPx3 (1×10^6^/100μL) was injected directly into the spleen after implantation of a tumor nodule on the surface of liver. Kinetic images of tumor growth *in vivo* were captured by Xenogen IVIS^®^ *in vivo* imaging system. Migrated hiPSC-MSCs in tumor tissues were detected by immuno-florescence (GFP) and IHC staining with anti-human fibroblast anti-body DIA100 (Dianova, Germany). Expression of GPx3 was detected by IHC staining with anti-human GPx3 anti-body (Abcam).

#### Statistical analysis

The Chi-square test was used to compare categorical data. The T test was adopted to compare continuous variables. For mRNA level of GPx3 within tissues, 2-fold difference was selected as cut-off point by which the patients could be segregated most evenly. Other cutoff points had also been taken into consideration and investigated. They showed the similar clinical implication. For plasma GPx3, The Youden index [42] was used to determine the optimal cutoff point for the prediction of five year recurrence. The cutoff point with the maximized Youden index showed the most optimal sensitivity and specificity for prediction. Kaplan-Meier survival analysis was performed to compare the survival outcome of HCC patients. The significant difference between survival outcomes was detected by the log–rank test. The Cox proportional hazard regression model was used to identify predictors for overall survival. The variables were selected into the final equation in multivariable analysis according to forward stepwise selection procedure. *P* < 0.05 was considered as statistically significant. Calculations were made using SPSS computer software version 16 (SPSS Inc, Chicago, IL, USA).

### SUPPLEMENTARY METHODS, FIGURES AND TABLES


