# Supplementary material for: Clinical significance and therapeutic value of glutathione peroxidase 3 (GPx3) in hepatocellular carcinoma
Source: Oncotarget. 2014 Oct 11;5(22):11103–20. doi: 10.18632/oncotarget.2549 (PMC4294380; doi:10.18632/oncotarget.2549)
Supplement: Supplementary file 1 [file oncotarget-05-11103-s001.pdf]

SUPPLEMENTARY METHODS, FIGURES AND TABLES

EMT detection

As rGPx3 showed obvious ability of anti-invasiveness, the role of rGPx3 in EMT process was detected. Expression of E-cadherin (Abcam), Vimentin (Epitomics, California, USA) were detected by western-blot with or without rGPx3 (Enzo Life Science, New York, USA) treatment (2μg/mL for 24h). As expression of Vimentin was hardly detected, EMT was induced in MHCC97L by pre-incubation with TGF-β (Cell signaling) at 20ng/mL for 48h to explore the effect of rGPx3 on EMT process. All the experimental condition was serum-free. In order to explore the down-stream targets, five E-cadherin transcriptional repressors, Slug, Snail, SIP1, Zeb1 and Twist were detected by qRT-PCR in MHCC97L under same conditions. The sequences of primers used were listed in Supplementary Table 1.

Regulation of NFκB signaling pathway

To explore whether the down-regulation of SIP1 was regulated by NFκB signaling pathway, the expression of SIP1 was detected in the MHCC97L pre-incubated with the NFκB inhibitor, pyrrolidine dithiocarbamate (PDTC, Sigma) at 60μM for 24h. To explore the role of rGPx3 in NFκB signaling pathway, NFκB nuclear translocation was detected by immunofluorescence in MHCC97L with or without rGPx3 treatment (2μg/mL for 24h pre-incubation). NFκB nuclear translocation in MHCC97L was stimulated by EGF (Peprotech Inc, Rocky Hill, NJ, USA) at 20ng/mL for 30min. After incubated with NFκB p56 rabbit-anti-human primary antibody (Cell Signaling) for 1h, MHCC97L was incubated with FITC conjugated goat-anti-rabbit secondary anti-body (Santa Cruz) for 30min and then detected under immunofluorescence microscope. Nuclear was counterstained with Dapi. All the experimental condition was serum-free.

Regulation of Erk signaling pathway

In order to investigate whether deactivation of NFκB by rGPx3 was regulated by Erk pathway, expression of Erk, p-Erk, NFκB-p65 and p-NFκB-p65 (all purchased from Cell Signaling) were detected in MHCC97L with or without rGPx3 treatment (2μg/mL for 24h pre-incubation) by western-blot. To explore whether activation of NFκB could be directly regulated by Erk signaling pathway, activation of NFκB were detected in MHCC97L stimulated by EGF (20ng/mL for 30min) with or without U0126 treatment (Erk inhibitor, 1,4-Diamino-2,3-dicyano-1,4-bis (o-aminophenylmercapto) butadiene, 10uM for 2h, Sigma). DMSO was used as delivery control. All the experimental condition was serum-free.

Regulation of MKP3 expression

In order to explore the deactivation of Erk by rGPx3 was regulated by MKP3, expression of MKP3 (Cell Signaling) were detected in MHCC97L treated with different concentration of rGPx3 for 24h. Erk were activated by EGF or HGF (Peprotech Inc, Rocky Hill, NJ, USA) at 20ng/mL or 40ng/mL for 30min in serum-free condition with or without pre-treatment of rGPx3 (2μg/mL for 24h) to investigate whether Erk activation was affected by rGPx3 treatment. To explore whether expression of MKP3 was determined by ROS level, expression of MKP3 was detected in MHCC97L treated with different concentration of H_2O_2.

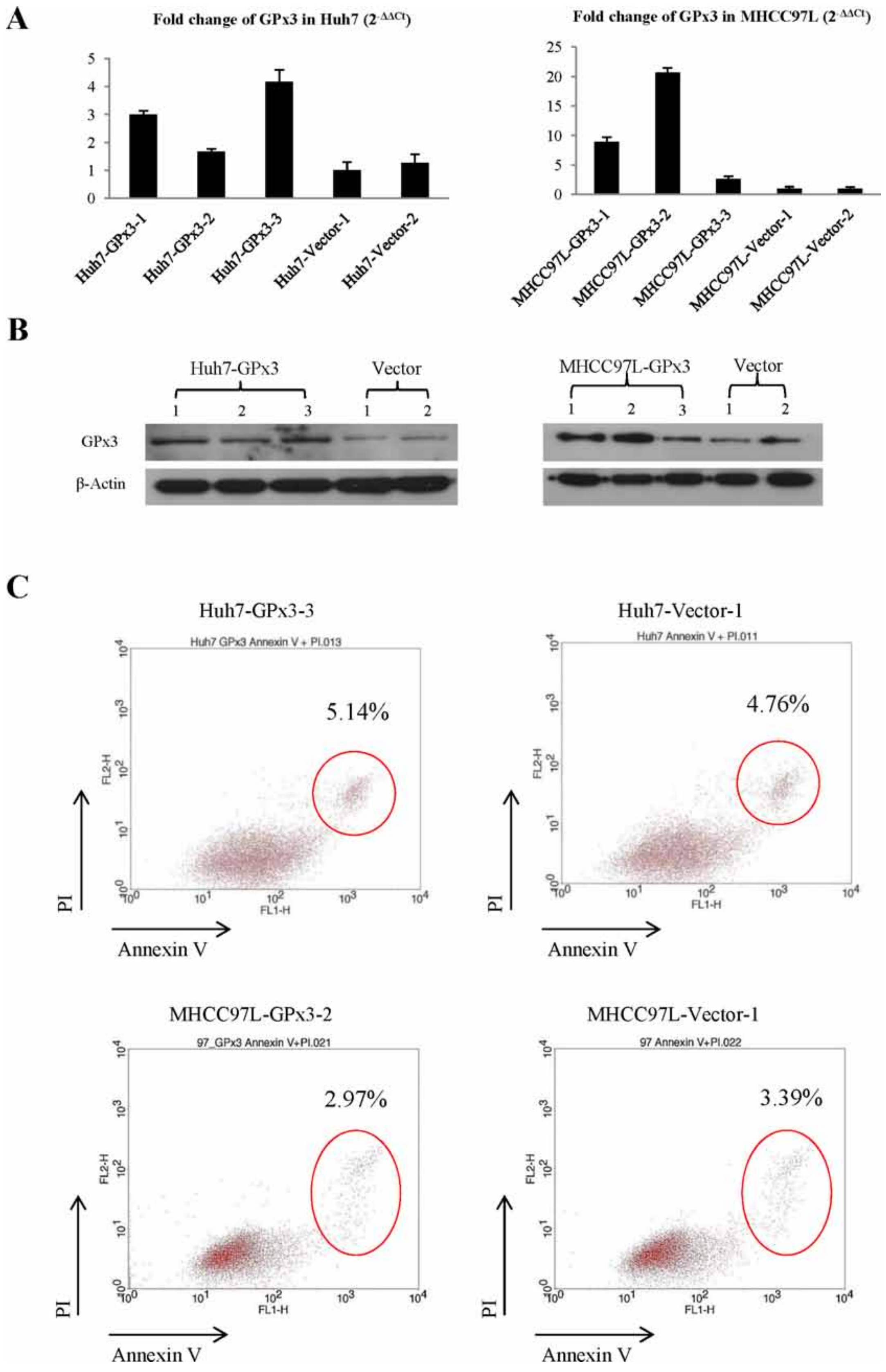

Supplementary Figure S1: Establishment of GPx3 stable transfectants and the effect of forced expression of GPx3 on apoptosis of HCC cells. **(A** and **B)** mRNA and protein level of GPx3 expression in HCC cells after transfection. **(C)** The proportion of apoptotic cells after transfection.

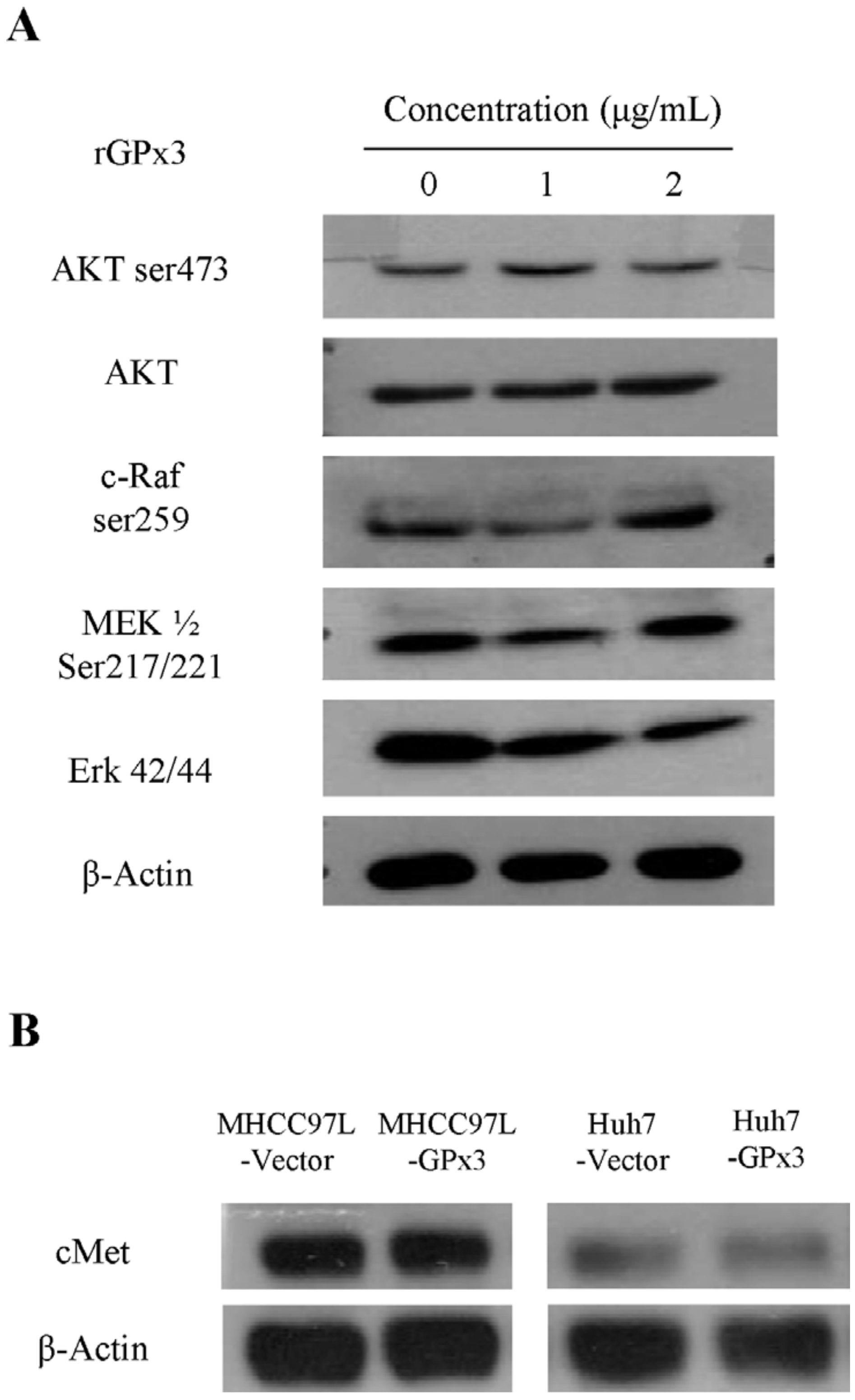

Supplementary Figure S2: Exploration of possible mechanism of tumor suppressive activity of GPx3 in HCC cells. **(A)** No change of activation of AKT was observed upon rGPx3 administration. Erk was deactivated upon rGPx3 administrationnot through Raf-MEK pathway. **(B)** No change of cMet expression was detectedupon rGPx3 administration.

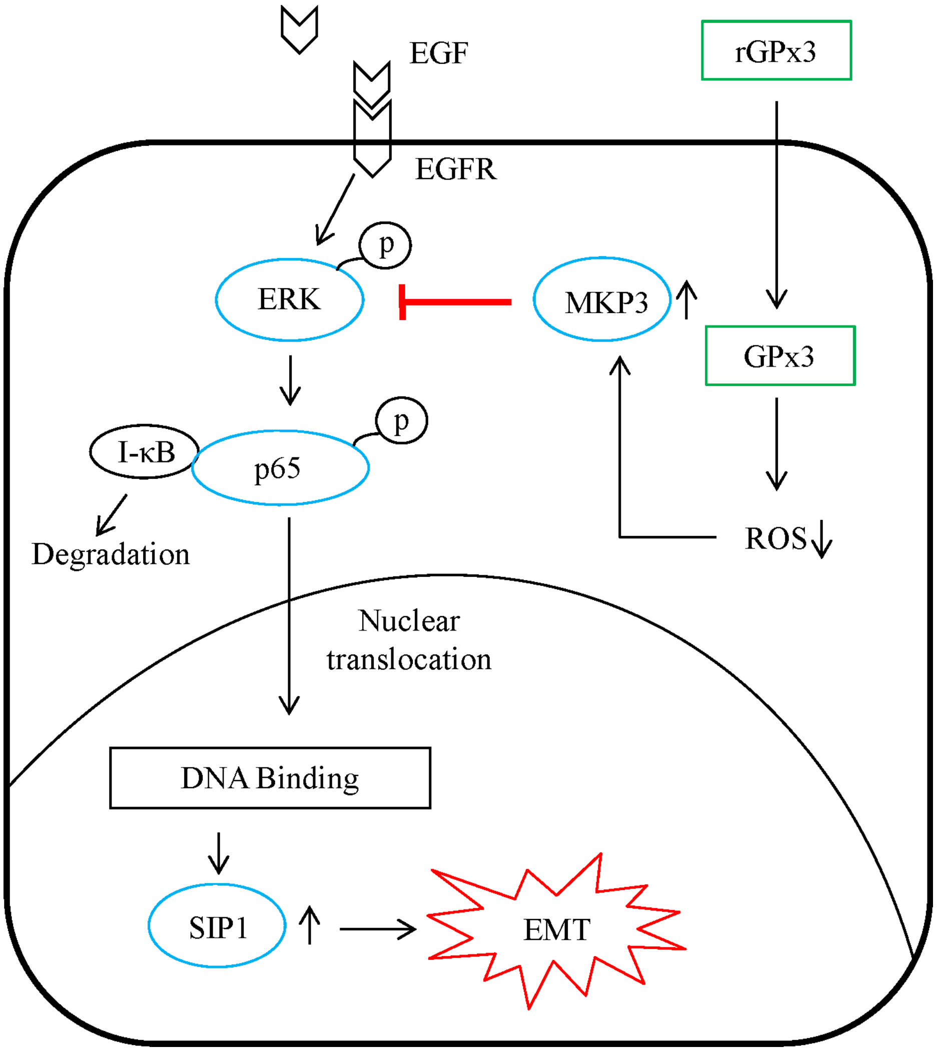

Supplementary Figure S3: The diagram showed the possible mechanism of tumor suppressive activity of GPx3.

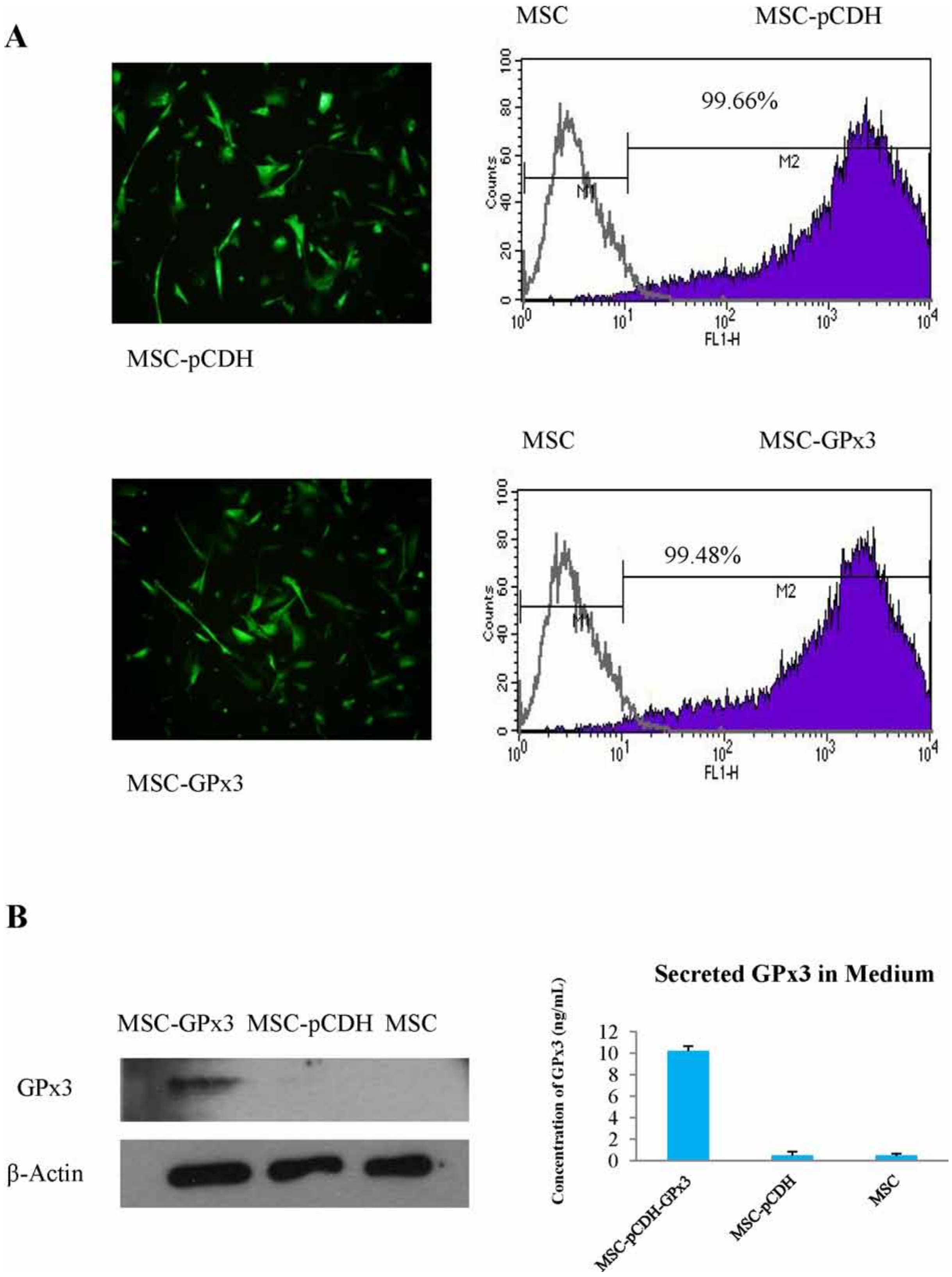

Supplementary Figure S4: Establishment of engineered hiPSC-MSCs delivering GPx3. **(A)** the transduction efficiency was nearly 100% detected by flow cytometry. **(B)** Protein level of GPx3 after transduction detected by Western-blot and ELISA.

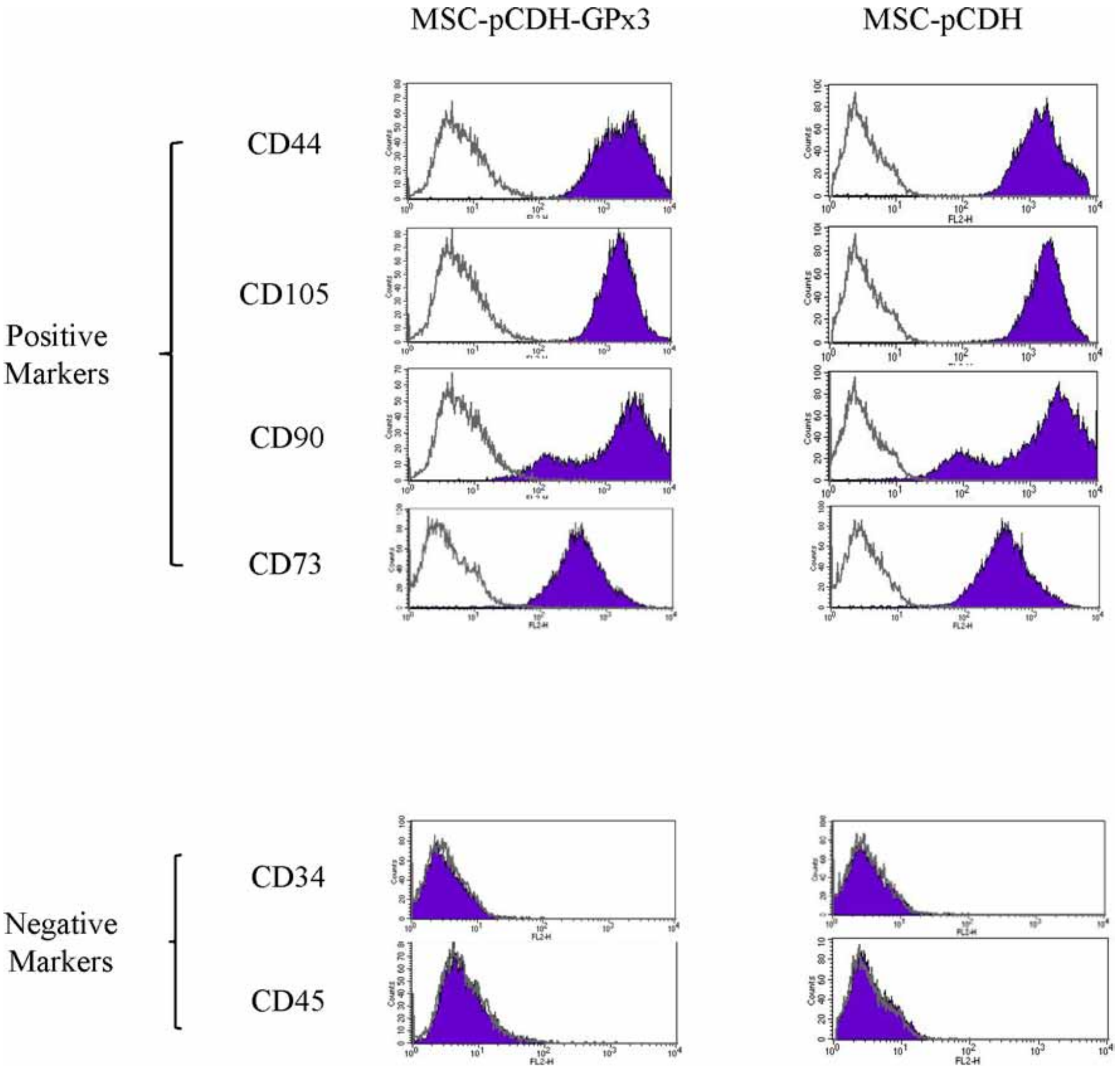

Supplementary Figure S5: Stem cells property could be maintained in hiPSC-MSCs after transduction of GPx3.

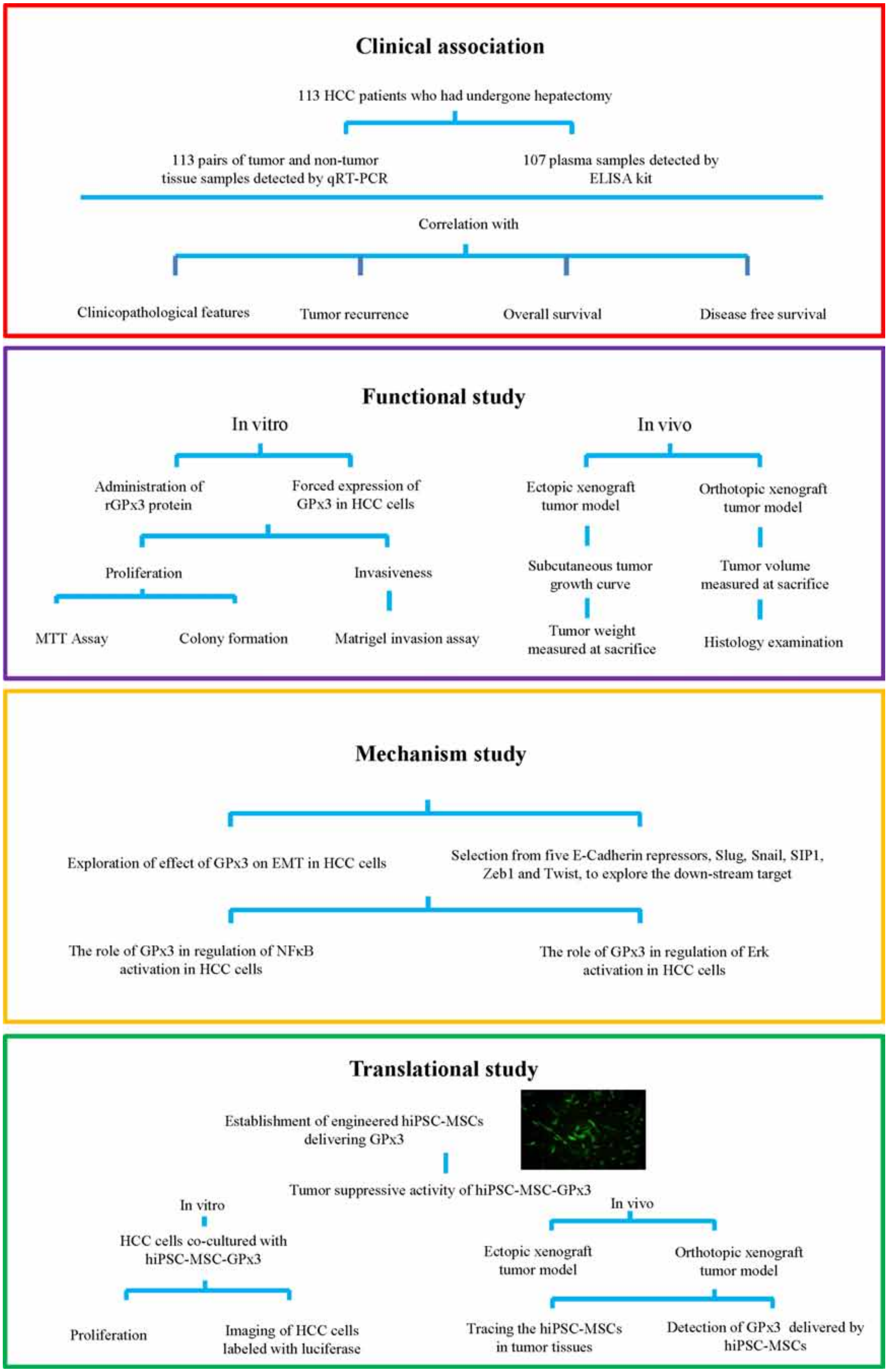

Supplementary Figure S6: The flow chart showed the whole study design.

Supplementary Table S1. Cox proportional hazard regression model for overall survival analysis

	Univariable analysis		Multivariable analysis	
	**HR(95%CI)**	***P***	**HR(95%CI)**	***P***
**GPx3 expression level**				
Down *vs* Non-down regulated	2.084 (1.209-3.593)	0.008	1.562 (0.906-2.693)	NS
**pTNM stage**				
Advanced *vs* Early	2.174 (1.344-3.516)	0.002	1.459 (0.502-4.242)	NS
**Venous infiltration**				
Presence *vs* Absence	4.692 (2.616-8.414)	0.000	3.003 (1.259-7.160)	0.013
**AFP level**				
>20 ng/mL *vs* ≤20 ng/mL	2.588 (1.510-4.436)	0.001	1.801 (0.995-3.260)	NS

Supplementary Table S2. Sequences of primer pairs

Gene name		Sequences
GPx3	Forward<br>Reverse	5′ GGGGATGTCAATGGAGAGAA 3′<br>5′ TTCATGGGTTCCCAGAAGAG 3′
Snail	Forward<br>Reverse	5′ GCCGCGCTCTTTCCTCGTCAG 3′<br>5′ AGCAGGTGGGCCTGGTCGTA 3′
Slug	Forward<br>Reverse	5′ TACCGCTGCTCCATTCCACGC 3′<br>5′ GACTGGGCATCGCAGTGCAG 3′
SIP1	Forward<br>Reverse	5′ GATGGCCCCCGGTGCAAGAG 3′<br>5′ CTGGATCGTGGCTTCTGGCCC 3′
Zeb1	Forward<br>Reverse	5′ GGCCCCAGGTGTAAGCGCAG 3′<br>5′ TGGTGTGCCCTGCCTCTGGT 3′
Twist	Forward<br>Reverse	5′ GCCGACGACAGCCTGAGCAA 3′<br>5′ CGCCTCGTTCAGCGACTGGG 3′
